# TCBLex - A lexical database of Finnish literary texts for children

**DOI:** 10.3758/s13428-025-02832-x

**Published:** 2025-10-15

**Authors:** Tapio Nojonen, Kiia Korsu, Filip Ginter, Veronika Laippala, Jenna Kanerva

**Affiliations:** 1 Department of Computing, University of Turku, Turku, Southwest Finland FI Finland; 2 Department of Biology, University of Turku, Turku, Southwest Finland FI Finland; 3 School of Languages and Translations Studies, University of Turku, Turku, Southwest Finland FI Finland

**Keywords:** Lexical database, Children, Finnish, Corpus, Frequency

## Abstract

This work introduces TCBLex, a lexical database of Finnish literary works read by children between the ages of 7 and 15. We explain in detail the work done to build the corpus TCBLex is based on, including how books were sampled and collected, turned into text files, and finally processed. We also touch on legal considerations and how it is possible to build such a corpus in the EU. TCBLex contains over 11 million tokens that are annotated with parts-of-speech tags and lemmatized. We provide 14 different sub-lexicons in total, covering individual intended reading ages, age groups, as well as different genres. We also provide versions with additional morphological information, such as the cases and tenses of words. TCBLex provides various psycholinguistically interesting lexical statistics for both word types and lemmas, such as different frequency metrics, distributions, word lengths, numbers of syllables, morphological paradigm sizes, and for the first time in a Finnish lexicon, ages when words and lemmas are first encountered in books. TCBLex is freely available at 10.5281/zenodo.15655580.

## Introduction

Reading is one of, if not the most, important skills a child has to learn in order to survive in the modern world. There has been research on how children’s reading abilities evolve from early childhood to the teenage years (see, e.g., Nurmi et al. (2014); Verhoeven and van Leeuwe (2008); Hulslander et al. (2010); Parrila et al. (2005)) and different models of reading have been proposed to explain this developmental process (Hoover & Gough, 1990; Joshi, 2019). Reading can positively affect the development of various skills and competencies, such as reading comprehension (Torppa et al., 2020) and writing proficiency (Jouhar & Rupley, 2021), and as such, it is important that children are shown texts they find interesting and that can encourage further reading.

At the same time, it is well known that mechanical reading will not improve literacy skills by itself (Nurmi et al., 2014) and for language learning in general, it is imperative that a reader understands enough of the contents of what they are reading for it to be useful (Laufer & Ravenhorst-Kalovski, 2010). Studies have also shown that children’s mental processing of text changes as reading skills develop (Grainger & Ziegler, 2011) and that the vocabulary children encounter can differ greatly depending on what age demographic a textual piece is written for (Korochkina et al., 2024).

As such, it is reasonable to assume that in children’s books, there are linguistic features, such as which words are most commonly used, that differ depending on what ages a book is recommended for (see, e.g. Dawson et al. (2023)). This kind of information is especially interesting when viewed through the lens of education, as teachers and writers of educational materials need to somehow curate and create suitable reading materials for students of different ages and with different levels of reading ability.

In order to gain scientific insight into what makes any given text suitable for particular age groups, information such as what kind of vocabulary is typical and what are common words is crucial. Also, from a psycholinguistics point of view, this kind of lexical information is invaluable for what types of words would act as good stimulus material (Schroeder et al., 2015). To create such a lexical database, there needs to be a corpus of children’s literary works, and these kinds of corpora have been made for various different languages (see e.g., Lété et al. (2004); Soares et al. (2014); Terzopoulos et al. (2016)). However, currently, none exist for Finnish.

Our research introduces the work done to create the first corpus of Finnish books for children. It is compiled from 300 books read by children aged 7 to 15 and has information on intended reading ages for each book. For the corpus, all books have been morphosyntactically analyzed with lemmas, syntactic roles as dependency relations, and various morphological tags. We also present a lexical database based on this corpus called the Turku Children’s Book Lexicon (TCBLex), which has more than 11 million tokens and over 9 million words. To our knowledge, this is the first Finnish lexicon to have detailed information on the age distribution of when words first appear in literary works and, e.g., how large a proportion of word types in books meant for children over 13 has been seen in books intended for younger children. We also provide additional statistics, such as the number of hapax words, the frequencies and Zipf values of words and lemmas, morphological paradigm sizes of lemmas, and proportions of part-of-speech (POS) tags. Due to copyright issues, we cannot release the whole corpus to the public, but we believe that TCBLex 1.0 still has great value for researching reading development of children in Finnish and for the broader psycholinguistics community.

## Background

There exists a lot of research on the development of children’s reading and other literacy skills (see Introduction). In corpus linguistics, much of the focus has been on foreign language learning (e.g., Li (2024); Bergström et al. (2022); Motabit (2020)) and children’s development of writing (e.g., Durrant (2022)). In the same vein, much of the corpus linguistics studies on what children read tend to only look at textbooks in relation to language learning and vocabulary (Khan, 2024; Van Parys et al., 2024), not reading development or how the language of the texts in them changes.

In the Finnish context, there are studies and corpora on texts written by children (for an overview of Finnish learner corpora, see Jantunen and Pirkola (2015)), but no such corpora exist for books written for children. However, as writing and reading are closely related to one another, these previous studies can still give a useful reference in what can be expected from texts meant for children. Perhaps the closest to our current study is a study by Pajunen and Honko on the analysis of schoolchildren’s essay writings using methods of corpus linguistics (Pajunen & Honko, 2021). The corpus in question has over 200,000 words and contains writings from each grade in the Finnish basic education system (grades 1–9, ages 7–15), as well as writings from young adults for the sake of comparisons (Pajunen, 2012). In this study, the authors found that the usage of very frequent words decreases with the writer’s age; that is to say that as children get older, their vocabulary size increases, and the dependence on very common words lessens.

Another finding that is particularly interesting is that the morphological paradigm sizes of lemmas (for a psycholinguistics definition, see, e.g., Lõo et al. (2024)) grow larger with age. (Pajunen & Honko, 2021). Finnish is a very different language compared to, e.g., English, as it is highly agglutinative and possesses 16 different cases and multiple suffixes which can be added to a lemma to gain increasingly complex and specific words (see, e.g., Korpela (2015)). As such, it makes sense that as writers get older, their ability to use complex morphological structures increases. This would naturally increase the paradigm size of most words. One final result worth highlighting here is that, although their research was not focused on syntactic analysis, they did find that in terms of Parts of Speech (POS) ratios, the older the writers, the more adjectives and nouns were used. Conversely, the ratio of verbs, pronouns, and conjunctions decreased. (Pajunen & Honko, 2021).

Although there is a clear difference between texts written by children and texts written for children by adults, these results can still serve as guiding posts. It would be reasonable to assume, for example, that the morphological paradigm size increases also happen in children’s books since, at least intuitively, it would make little sense for morphologically more complex language to appear more often in texts meant for less skilled readers.

### Previously compiled corpora of children’s literature

**Table 1:** Corpora of children’s books with focus on how book language read by children evolves

Name	Language	Size	Number of books	Ages included	Authors	Release year
MANULEX	French	2 million words	54	6–11	Lété et al.	2004
OCC	English	30 million tokens	NaN	5–14	Wild et al.	2013
ESCOLEX	Portuguese	3.2 million tokens	171	6–11	Soares et al.	2014
BasiLex	Dutch	13.5 million tokens	NaN	4–12	Tellings et al.	2014
childLex	German	10 million tokens	500	6–12	Schroeder et al.	2015
HelexKids	Greek	$\approx$1.4 million tokens	116	6–12	Terzopoulos et al.	2016
CCLOWW	Chinese	22 million word tokens	2131	7–12	Li et al.	2023
CYP-LEX	English	70 million tokens	1200	5–18	Korochkina et al.	2024

NaN means that no explicit amount of books is given and that the corpus also includes textual material from other sources

Previously compiled corpora of children’s books can be categorized into those that focus solely on textbooks and educational materials, including ESCOLEX for Portuguese (Soares et al., 2014) and HelexKids for Greek (Terzopoulos et al., 2016), as well as the rest, which are far broader in scope and contain texts meant to be read in leisure time in addition to purely educational materials. This means both fictional works like novels as well as some non-textbook, nonfiction books, like cookbooks. However, genre was not an important part in book selection, and mainly non-educational books mean fictional works (Korochkina et al., 2024; Schroeder et al., 2015).

As can be seen from Table 1, the sizes of previously compiled children’s corpora vary from low millions to high tens of millions of tokens, but especially notable is how those corpora consisting of purely educational materials are far smaller in size compared to the other works. Regardless of size or what genre of books was used in building the corpus, the main goal of each study that introduced one of these corpora has been to provide insight into the lexical richness of the writing encountered by children. Some studies have focused more on directly comparing the differences between the vocabulary encountered by children and adults (Wild et al., 2013; Tellings et al., 2014; Li et al., 2023; Korochkina et al., 2024), while others put more emphasis on statistics found in these children’s lexical databases (Lété et al., 2004; Soares et al., 2014; Schroeder et al., 2015; Terzopoulos et al., 2016).

For the sampling of books to include in a corpus, different studies have chosen different methods. Broadly speaking, we found three main sources from which books were sampled into corpora. The first and most commonly mentioned of these was commercial book retailers and different sales rankings. Both childLex and CYP-LEX used, for example, Amazon, as it provides lists of the most sold children’s books over different spans, such as weekly and annually (Korochkina et al., 2024; Schroeder et al., 2015). CCLOWW, in turn, used the two most popular Chinese book retailers and their annual sales rankings (Li et al., 2023) and MANULEX based its sampling on sales figures released by publishers (Lété et al., 2004).

The second source included various reports and surveys. In the creation of CYP-LEX and childLex, the authors used the findings of different national surveys to find books that were popular among children (Korochkina et al., 2024; Schroeder et al., 2015). Additionally, childLex used loan statistics from the State Library of Berlin.

The third source was schools and official documents. This category includes school library recommendations from government agencies (Li et al., 2023), textbooks mandated by curricula (Terzopoulos et al., 2016), and textbooks recommended by schools and publishers (Schroeder et al., 2015; Soares et al., 2014).

Most of the corpora focused solely on children in their respective countries’ primary schools, so typically grades 1–6. The exceptions to this are CYP-LEX and OCC, which also included books for children in secondary school (aged 13 and over) (Korochkina et al., 2024; Wild et al., 2013), as well as BasiLex, which included some books for children in the first two years of secondary school (Tellings et al., 2014).

These previously done corpora also differ in how they decided to split books into groups. ESCOLEX, HelexKids, and CCLOWW did not do groupings, so each grade acted as its own category (Soares et al., 2014; Terzopoulos et al., 2016; Li et al., 2023). MANULEX and BasiLex contain both groups that span multiple grades as well as individual grades as their own groups. In the case of MANULEX, grade 1 and grade 2 were separate groups, but grades 3–5 were grouped into one (Lété et al., 2004). For BasiLex, primary school grades were each their own grouping, but kindergarten and secondary school contained more than one grade per group (Tellings et al., 2014). Finally, OCC, childLex, and CYP-LEX used age groups instead of grades for their groupings, with the English using groupings based on Key Stages 1–3 defined in the British National Curriculum (Department of Education, 2014), and childLex opting for three different age groups: 6–8, 9–10, and 11–12. (Wild et al., 2013; Schroeder et al., 2015; Korochkina et al., 2024).

There are also major differences in how much material from each age group was included. In general, this is because books for older children tend to be longer than books for young children (Korochkina et al., 2024) and because different studies chose different sampling methods. For example, in childLex, the authors decided to include an unequal number of books and tokens from each age group, resulting in the youngest age group containing nearly three times the number of books when compared to the oldest age group. The main reason for this was that they wanted to have “a sufficient number of words in each age group”, which led to the oversampling of books from younger age groups. (Schroeder et al., 2015). This is in stark contrast to CYP-LEX, where the authors decided to have an equal amount of books per age group, noting that it would not make for a representative corpus if they tried to oversample books to level the token amounts – after all, older children can likely read longer books on average and the amount of words they encounter is greater (Korochkina et al., 2024). In general, there is no unifying or widely adopted methodology that was used in previous works to determine how many books or words should be aimed for per category. Also, none of the previously made corpora decided to equal the token amounts between all age groups.

### Statistics reported in previous studies

Generally, statistics reported in previous studies alongside the basics, such as the number of words, include frequency metrics for words and lemmas, some type of dispersion metric, and POS tag frequencies (Lété et al., 2004; Wild et al., 2013; Tellings et al., 2014; Soares et al., 2014; Schroeder et al., 2015; Terzopoulos et al., 2016; Li et al., 2023; Korochkina et al., 2024). Traditionally, the golden standard for frequency metrics was the trio of overall word frequency (F), estimated frequency per million words (U) and standardized frequency index (SFI) (Lété et al., 2004; Wild et al., 2013; Soares et al., 2014; Schroeder et al., 2015). Recently, reporting has shifted to favor Zipf values over U and SFI, as the Zipf scale is seen as a better way of calculating and representing standardized frequencies (see van Heuven et al. (2014)). For CCLOWW and CYP-LEX, U and SFI were completely omitted (Li et al., 2023; Korochkina et al., 2024) and for HelexKids, which wanted to retain easy comparability with older works, the authors reported U and SFI alongside Zipf values (Terzopoulos et al., 2016).

For distribution metrics, the previous studies reported mainly contextual diversity (CD) (Li et al., 2023; Soares et al., 2014; Terzopoulos et al., 2016), dispersion (D) (Lété et al., 2004; Soares et al., 2014; Schroeder et al., 2015; Terzopoulos et al., 2016), or simply inverse document frequency (IDF) (Korochkina et al., 2024). All of these measures essentially give a numerical value to how many different contexts a word type appears in. For example, two words might have the exact same absolute frequency of 500 in the corpus, but there is a clear difference if one of them appears 500 times in a single book or if it appears once in 500 books. In fact, some studies have argued and shown that these distribution measures matter more than raw frequency counts when it comes to assessing language development (Adelman et al., 2006).

Beyond these basic statistics, many of the previous works also reported various kinds of data which is especially relevant to psycholinguistics. The most common of these statistics include the average length of word types in characters and syllabic information, such as the number of syllables per word type (Schroeder et al., 2015; Soares et al., 2014).

Some of the key findings that are consistent among these previous studies are that the average length of word types tends to increase with target age (Schroeder et al., 2015; Lété et al., 2004), that there are clear differences in terms of vocabulary between books for certain ages and that many of the words children encounter might be age or age group specific (Schroeder et al., 2015; Korochkina et al., 2024; Soares et al., 2014), and that a large part of the words in children’s books are so called hapax words, meaning word types which occur only once (Schroeder et al., 2015; Li et al., 2023; Soares et al., 2014; Terzopoulos et al., 2016; Lété et al., 2004).

## Methods

### Sampling for TCBLex

We chose to initially gather a list of books for which we could reliably get their intended reading ages and then heuristically narrow it down based on the categories outlined in previous works. Some options mentioned in previous studies, such as using Amazon for book sales rankings, were unavailable for Finnish. Similarly, we did not have access to any book-level loan statistics from libraries. Thus, we decided to start by gathering a list of all the fiction, nonfiction, and educational children’s books displayed on the largest book retailer in Finland: Suomalainen Kirjakauppa (Kirjakauppa, 2025) as well as their intended reading ages. For Suomalainen, intended reading ages were provided as exact ages. For the books that did not have this information, we searched them on other retailers’ and publishers’ websites and added their target ages manually. If an intended reading age was given as an age span (e.g., 8–11), we chose the youngest age within the span. If an intended reading age was not found, that book was discarded from the list. This resulted in a list of over 3400 books intended to be read by children in grades 1–9.

For age groups, initially we separated all the books into three age groups akin to childLex, CYP-LEX, and OCC (Wild et al., 2013; Schroeder et al., 2015; Korochkina et al., 2024). We decided on ages 7–8 being age group one, ages 9–12 being age group two, and ages 13 and up being age group three. These were chosen based on the Finnish Basic Education Guidelines in the most recent National Curriculum Guidelines for Basic Education (Opetushallitus, 2014), as the guidelines draw clear differences in Finnish education between grades corresponding to these ages (grades 1–2, grades 3–6, and grades 7–9). We also kept track of the exact intended reading ages of the books, since the sources used in gathering the list of books provided this detailed information. This extra information was used to see how books are distributed within our chosen age groups, as well as to keep track of when word types are first seen. We did not use this information about the exact recommended reading ages for sampling the current version of our corpus, but this might be taken into account in the future.

After separating the books into their respective age groups, we sorted the books by their sales rankings on Suomalainen Kirjakauppa. We also used the statistics by Kirjakauppaliitto (Kirjakauppaliitto, 2025), whose monthly lists show the ten best-selling children’s books in Finland. We then decided to give even higher priority to books mentioned as the most popular ones in a national study called Lukuklaani, which surveyed the reading habits of Finnish students (Satokangas et al., 2022). Additionally, based on the results of Lukuklaani, we decided to include some books meant for children between the ages of 5 and 6 in our sampling, as some of the most popular books for children in the first years of basic education were intended to be read by younger children. Finally, the yearly sales rankings published by Suomen Kustannusyhdistys (Bestsellerit, 2025) were taken into account and used to give priority to those books that performed well in sales over the past 14 years.

After sorting the books in terms of priority, we created additional sampling guidelines to control which books were included. As has been found by other researchers (e.g., Gardner (2008)), factors such as theme and author can have a noticeable impact on the vocabulary of literary works. Thus, we decided to limit the number of books by each author to ten. Similarly, we decided to limit the number of books per book series by the same author to three. While it is true that popular series can make up a significant portion of a child’s leisure reading, such as Harry Potter, we thought that it was better to focus on the breadth of what children’s books have to offer.

We did, however, decide to take into account different genres, as we wanted to include nonfiction works in the corpus, even if they were not high up in any sales rankings. For this, we analyzed the loan statistics of Finnish libraries for the past 20 years. As mentioned before, we did not have access to book-level data, but the National Library of Finland provides publicly available statistics on how many books per category are loaned out in the whole country each year, and found that children’s fiction books were loaned out around eight times more often than nonfiction ones. (Kansalliskirjasto, 2025). While these loan statistics might not be perfectly representative, as children also read a lot of textbooks which are mostly nonfictional, we decided to use them as a guideline such that fictional works should be multiple times more numerous than nonfiction books. For TCBLex 1.0, the ratio between fiction books and nonfiction books (further separated into textbooks and other nonfiction) is 3:1.

During the collection of the books, we tried our best to adhere to our sampling guidelines and heuristics. One of the biggest issues was that some of the most popular books in terms of sales had significant availability issues. We chose to proceed in the same manner as what was reported in the papers presenting childLex and CCLOWW - if a book was unavailable to us, we simply chose the next best option while keeping in mind the other restrictions to maintain correct ratios (Schroeder et al., 2015; Li et al., 2023).

### Legal considerations for the compilation process

One of the main hurdles for this study was how to obtain all the works needed for compiling a truly representative corpus of children’s Finnish literary works, as these would all be under copyright. For example, in the creation of CYP-LEX, the authors either purchased e-books or used books that were no longer under copyright (Korochkina et al., 2024). This was not possible in our situation, as we did not have the necessary funding to purchase hundreds of e-books, nor did there exist a substantial amount of relevant Finnish books not under copyright. Thus, we decided to follow the approach used in the creation of, e.g., MANULEX and childLex - physically scanning the necessary books by taking pictures and using optical character recognition (OCR) software to transform the images into text (Lété et al., 2004; Schroeder et al., 2015).

Under the so-called textual data mining (TDM) exception, research organizations are allowed to make copies of textual materials they have legal access to, use this material for textual and data mining so long as it is for scientific purposes, and that this cannot be prevented by technical means (EU Directive 2019/790; Tekijänoikeuslaki 1961/404 13 b §)(https://www.finlex.fi/fi/lainsaadanto/1961/404, https://eur-lex.europa.eu/eli/dir/2019/790/oj/eng). For the scanning process, under the TDM exception, we decided to utilize the services of local libraries, as they give legal access to a wide variety of books.

### Processing pipeline

The process from obtaining the books to a ready corpus consisted of the following steps. First, physical books were photographed using a high-resolution document camera as JPEGs, which were then transformed into PDF files. We chose to omit pages not very relevant to the reading contents of the book, such as publishing information and acknowledgments. These PDF files were then processed using Google Cloud Services’ (GCS) Document AI Layout Processor. Initially, we used Tesseract OCR software (Ooms, 2025), but after some test runs, we switched to GCS as the OCR performance seemed much better. Additionally, Layout Processor allowed us to filter out much of the noise resulting from certain children’s books, such as pictures containing texts placed in the middle of paragraphs. After obtaining the layouts and turning them into raw text files, we manually cleaned the texts as there was a fair amount of noise from the OCR process. For example, Finnish uses umlauts, and the letter ‘Ä’ would sometimes get wrongly interpreted as ‘A’ or even ‘Ã’. After manual cleaning, the texts were processed with the Trankit syntactic parser (Nguyen et al., 2021), using a Finnish model trained on the UD Finnish TDT treebank (Pyysalo et al., 2015) following the Universal Dependencies (UD) annotation scheme (de Marneffe et al., 2021; Nivre et al., 2020). Trankit for Finnish using the TDT treebank achieves over 99.6% accuracy for tokens, around 98% accuracy in POS tagging and around 95% accuracy for lemmatization (Trankit documentation) (https://trankit.readthedocs.io/en/latest/performance.html). Since we used Trankit with TDT, all UD POS tags except DET and PART are used (for more information on POS tags, see Pyysalo et al. (2015)).

As mentioned before, since we are using copyrighted material in our research, we are unable to release the whole corpus, which contains unedited paragraphs with syntactic information. However, this is not an issue for a lexical database, which is why we can release TCBLex to the public, and going forward in this paper, we will exclusively talk about this lexical database.

TCBLex 1.0 is released as four collections of CSV files; one contains information on exact age level, one contains information on age group level, one contains information on the book genre level, and the final one has data that encompasses the whole lexicon. There is also another set of files, which contain versions of the sub-lexicons with additional information on the features of the words for those especially interested in Finnish morphology. These additional features include, for example, the mood and case of the word. In this paper, we exclusively refer to versions without additional features. On the GitHub page, we also provide the code that has been used to calculate the numbers presented in this paper.

### Statistics provided in TCBLex

In our reporting, we present various statistics of TCBLex in terms of tokens, word types, and lemmas. We use the term tokens instead of words to highlight that TCBLex contains a number of non-words, such as full stops and periods. With word type, we mean a token with a specific morphological form, which can occur multiple times in the corpus. For example, the word type “tulen” in Finnish can occur as a noun in the genitive case (“fire’s” in English) or a verb (“I will come” or “I am coming” in English). The lemma, then, is the base form of a word type informed by, for example, the POS tag. In this example, the lemma would be “tuli” if referring to a noun (“fire” in English) or “tulla” if referring to a verb (“to come” in English).

For the files that make up TCBLex, each one starts with three columns, which mark the word type, the lemma, and the POS tag. For POS tags, we have chosen to use the “upos” tags given by Trankit (de Marneffe et al., 2021). These three columns are then followed by different kinds of numerical statistics, such as the length of the word or the lemma in terms of characters. These statistics hold within the sub-lexicon specified in the name of the CSV file, so the same word type might have different statistics depending on the sub-lexicon.

#### Raw frequency (F)

Marks the absolute frequency of the specified entity inside the sub-lexicon. Word F and Lemma F mark the frequencies of the specified word or lemma types. Then Word-POS F marks the frequency of the bigram consisting of a word type and a POS tag. As the same word type can have multiple different meanings in Finnish, this bigram frequency gives insight into how commonly a word is used, e.g., as a main verb or as an auxiliary verb. It should be noted that, for this reason, it can be very difficult to determine whether entries in the lexicon with high Word F and low Word-POS F are rare bigrams with correct annotations or parsing mistakes that Trankit infrequently makes (see the Processing pipeline section).

#### Contextual diversity (CD)

Marks the contextual diversity value of the word type or the lemma. CD is calculated simply by taking the number of books in the sub-lexicon where the word or lemma appears and dividing by the total number of books in the sub-lexicon. As such, the numbers cannot be directly compared to one another between sub-lexicons. For example, if a word appears in only one book in the whole lexicon and that book has an intended reading age of 12, then the CD value for said word would be $1/100 = 0.01$ in the ’9-12’ sub-lexicon and $1 / 300 \approx 0.003$ in the whole lexicon.

#### Dispersion (D)

Marks the dispersion value of the word type or lemma. Dispersion is calculated with the formula: $D = \{\log (\sum p_i) - [\sum (p_i \log p_i) / \sum (p_i)]\} / \log (n)$, where *n* is the number of books in the sub-lexicon and $p_i$ the frequency of a word/lemma in book *i*. Dispersion is 0 if a word occurs only once and in only one book, and has a maximum value of 1.

#### Estimated frequency per 1,000,000 words (U)

Marks the estimated frequency of the word or lemma per 1,000,000 words, adjusted for *D*. When $D=1$, which means that the word occurs very frequently in all books, then *U* is equal to the frequency per 1,000,000 words. If $D<1$, which it almost always is, then the value is adjusted downward to account for the fact that the word is not necessarily commonly seen in different books. The complete formula for *U* is: $U = (1,000,000/N)[F * D + (1 - D) * f_{min}]$, where *N* is the total number of words in the sub-lexicon and $f_{min}$ is a scaler given by the formula: $f_{min} = (1/N)*\sum (f_i * s_i)$, where $f_i$ is the frequency of the word in book *i* and $s_i$ the total number of words in book *i*.

#### Standardized Frequency Index (SFI)

Marks the Standardized Frequency Index of a word or lemma. It is directly derived from *U* by standardizing it on a $log_{10}$ scale, with the reference value being 40, which equates to the word/lemma appearing once per million words. The formula is as follows: $SFI = 10 * [log_{10}(U) + 4]$. So, for example, if a word has $SFI = 80$, then the word is expected to appear once in 100 words, and if $SFI = 70$, then the expectation is to encounter the word once in 1000 words.

#### Zipf

Marks the Zipf value of a word or lemma. The Zipf value ranges from around 1, meaning extremely low frequency, to around 7, meaning very high frequency. Most low-frequency words have values around 2.5, and common words have values around or above 4.5 (van Heuven et al., 2014). The formula for calculating the Zipf value of a word for any given (sub)lexicon is: $Zipf = \log _{10}(\frac{F + 1}{n_i + t_i})+3$, where $n_i$ is the total number of words divided by 1,000,000, and $t_i$ is the total number of word types divided by 1,000,000. The Laplace transformation is used to account for words that might have a frequency of 0 (van Heuven et al., 2014).

#### Word syllables

Marks the number of syllables in the word. Syllable counts have been automatically computed following the most common rules for Finnish. Words that start with punctuation or a non-alphabetic character have a syllable count of 0 and otherwise follow common rules for written, standard Finnish. All words have been processed with the same rules, so note that, e.g., words from some dialect of Finnish might display a different amount of syllables than if they were processed with rules for said dialect.

#### Morphological paradigm size (MPS)

Marks the morphological paradigm size of the lemma. This is calculated by counting the number of different word-lemma bigrams with the same lemma, which gives the number of different inflections for any given lemma inside a sub-lexicon. So, if a lemma has $MPS = 1$, that means that the lemma is always encountered only in a particular form given by the word type, whereas $MPS = 100$ means that there are 100 different word types that have been lemmatized to the same lemma.

#### FSA

Marks the intended reading age when a word type or lemma is first encountered. These values carry over from one sub-lexicon to another, meaning that if some word like “ja” (Finnish for “and”) is naturally first encountered in a book with the intended reading age of 5, then the FSA value will always be 5, regardless of which sub-lexicon you are looking at. So, if a word has $FSA=13$, then that means that the word is not seen even once in books with a lower intended reading age.

To our knowledge, this type of data does not yet exist for Finnish, and while it is not directly comparable to age of acquisition, it gives insight into how the lexical make-up of books evolves and, for example, how word types carry over as the intended reading age increases.

**Table 2:** Sizes of the lexical databases for each age specific sub-lexicon

	Tokens	Word types	Hapax words	Lemmas
Age 5	264,916	51,893	34,664	23,619
Age 6	359,346	58,280	36,711	23,782
Age 7	805,586	105,176	64,827	42,854
Age 8	956,309	110,369	67,235	44,150
Age 9	1,762,154	166,520	97,659	63,513
Age 10	502,469	74,495	46,821	30,202
Age 11	299,504	53,351	33,208	22,794
Age 12	1,401,351	146,708	88,855	55,921
Age 13	935,010	122,702	76,161	49,149
Age 14	2,542,212	219,122	129,892	83,816
Age 15	1,764,020	182,214	109,824	70,213

## Results

TCBLex 1.0 has the texts of 300 books read by children between the ages of 7 and 15. Each age group (’7–8’, ’9–12’ and ’13+’) contains a hundred books, of which 75 are fiction books, ten are textbooks, and 15 are other nonfiction books. Each book has been fully included, meaning that TCBLex contains all words present in the scanned pages. When looking at the exact intended reading ages, the distributions of word amounts inside sub-lexicons are not equal (Table 2).

**Fig. 1:**
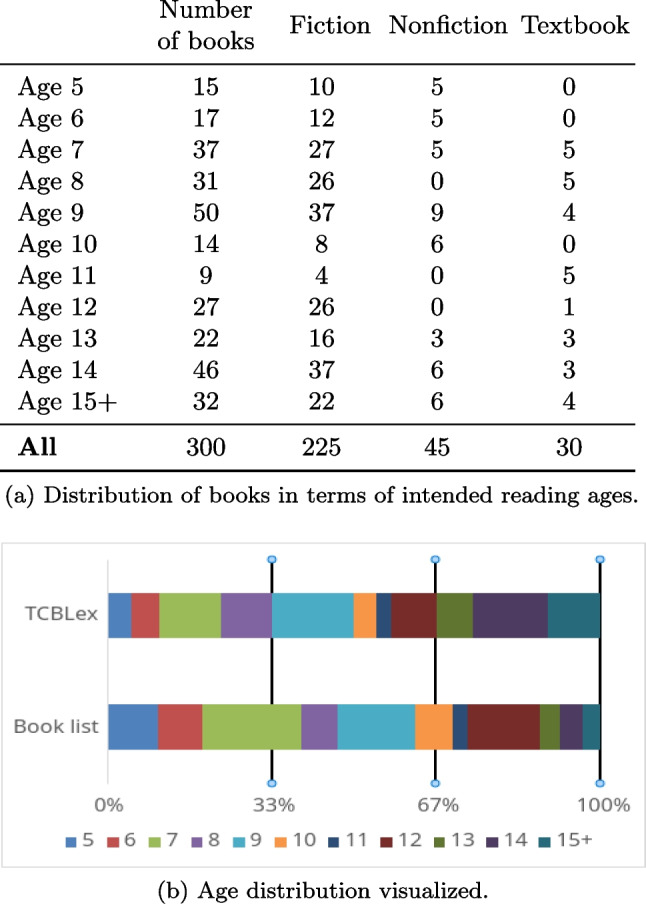
Comparing the age distributions of TCBLex 1.0 and the whole list of books TCBLex is sampled from

This is to be expected, since we sampled the books based on age groups rather than individual ages. If we look at the distribution of books in TCBLex 1.0 and the list of books we sampled from, one can see that the intended reading ages are not equally distributed in the original list (Fig. 1b). Thus, when we sampled books from the age groups, this naturally led to there being a higher chance of a book getting picked from an age group that had more books within the age group. We also wanted to sample such that each age group had exactly as many books as the other. This led to more words for older ages, which is similar to what was observed in CYP-LEX, which also used similar sampling guidelines (Korochkina et al., 2024).

One must also bear in mind that these exact ages are not necessarily reliable. There is no publicly available data on how reading ages are assigned, and as such, there is no guarantee that the amounts in our book list correspond to there being, for example, many more books for seven-year-olds compared to other ages. Although the site we mainly used provided exact ages, nearly all other retailers and sources mentioned in previous papers (e.g., Schroeder et al. (2015)) provide intended reading ages as spans. So, while TCBLex 1.0 contains age-specific information, the statistics are intended to be looked at first and foremost in age groups (Table 3). We also caution against making drastic conclusions when comparing statistics between two age-specific sub-lexicons, as they might vary greatly in size and what genre of books are included (Fig. 1a).

In terms of size, TCBLex 1.0 contains nearly 11.6 million tokens in total. From these, there are almost 620,000 different word types, of which close to 357,000 are so-called hapax words, meaning that they occur only once. The number of lemmas at around 243,000 is predictably lower than the number of word types (Table 3). Compared with previously compiled corpora of children’s literature, the word type and lemma amounts seem reasonable. Especially when looking at childLex and BasiLex, which are the closest to TCBLex 1.0 in size, the number of word types and lemmas is quite similar (Schroeder et al., 2015; Tellings et al., 2014).

**Table 3:** Sizes of the lexical databases for each age group as well as the whole of TCBLex 1.0

	Tokens	Word types	Hapax words	Lemmas
Age 7–8	2,386,157	221,327	132,937	88,358
Age 9–12	3,965,478	297,538	172,221	112,894
Age 13+	5,241,242	367,494	214,054	140,515
All	11,592,877	620,614	356,779	243,332

Although the amount of word types and lemmas is greater in TCBLex, we can potentially attribute at least the high number of word types to a fairly natural reason; since Finnish is a very inflectional and morphologically complex language, the number of word types relative to the token count being high in comparison to other languages is intuitive (see, e.g. Kettunen (2014)). One technical reason for the number of lemmas might be related to the high number of word types. As the parser we used is not perfectly accurate in lemmatization for Finnish (see the Processing pipeline section), this can potentially lead to a relatively high number of lemmatizing mistakes, of which most produce an entry that only occurs once in the lexicon, thus inflating the number of lemmas.

TCBLex 1.0 also seems to continue the trend of children’s books having a large proportion of hapax words. This was observed all the way back in the paper presenting MANULEX, where 31% of all word types occurred only once (Lété et al., 2004). The rate of hapax words changes depending on the language, but in previous studies it has ranged from around 20–25% (Soares et al., 2014; Li et al., 2023) up to around 45–50 % (Terzopoulos et al., 2016; Schroeder et al., 2015). By contrast, the rate of hapax word types for TCBLex 1.0 is around 57.5%, which is higher than any of the previously reported proportions. The rate of hapax lemmas (lemmas occurring only once) is also very high, reaching nearly 56 % for the entire lexicon. Although the proportions are clearly higher for TCBLex 1.0, this trend seems to be in line with childLex, where the proportion of hapax words is around 48.7% and the proportion of hapax lemmas is slightly lower at 48.3% (Schroeder et al., 2015). However, it should be noted that the ratio of hapax lemmas is likely lower, as some of the hapax lemmas are undoubtedly the result of lemmatizing mistakes.

**Table 4:** Sizes of the lexical databases for each genre as well as the whole of TCBLex 1.0

	Tokens	Word types	Hapax words	Lemmas
Fiction	10,142,893	514,182	292,582	195,892
Nonfiction	942,972	159,091	102,369	68,222
Textbook	507,012	86,053	52,334	38,186
All	11,592,877	620,614	356,779	243,332

For different genres, clearly the main source in both books and words is fiction (Table 4). Although textbooks and other non-fiction books account for a fourth of the books in TCBLex 1.0, they only make up around 12.5% of the tokens. This discrepancy is mainly due to the fact that there are differences in the average length of books depending on the intended reading age and genre. For the youngest age group, the average token counts for fiction, nonfiction, and textbooks are about 28k, 11k, and 15k, respectively. The counts for the oldest age group, on the other hand, are around 61k, 33k, and 17k, respectively. From these averages, we can see that while textbooks are about as long for both age groups, books meant to be read in leisure time seem to grow significantly longer, as intended reading age increases. And, as can be seen from the averages, fictional works tend to be a lot longer than books in the two other categories, which then leads to the token counts being disproportionate to the book proportions.

*Syntactic categories* In terms of syntactic categories, there are some differences between age groups found in TCBLex. It would seem that the number of nouns decreases as the intended reading age increases, while the number of proper nouns decreases initially and then stabilizes for the second and third age groups. In turn, auxiliary verbs and pronouns seem to increase with the intended target age (Table 5.) In childLex, similar trends were found. For example, noun tokens were observed to become less common with an increase in target age, going from around $17.25\%$ for ages 6–8 to about $16.45\%$ for ages 11–12. For another example, pronouns were found to go from around $12.45\%$ for 6–8 to about $13.82\%$ for 11–12. (Schroeder et al., 2015). So, while the proportions are different between our observations and those in childLex, the trends seem to be similar and of similar magnitude. However, our findings are contradictory to those observed by Pajunen and Honko (Pajunen & Honko, 2021), where, e.g., the amount of nouns was seen to increase as the ages of writers increased. This might be due to differing datasets (texts written by students compared to books written for children by adults), but further research is needed.

**Table 5:** Percentages of POS-tags in terms of tokens for the entire TCBLex 1.0

	All	7–8	9–12	13+
ADJ	5.2	5.6	5.1	5.2
ADP	1.4	1.4	1.4	1.4
ADV	9.0	9.1	9.2	8.9
AUX	7.0	6.4	6.8	7.1
CCONJ	4.0	3.9	3.8	4.0
INTJ	0.4	0.4	0.3	0.4
NOUN	18.8	19.5	19.1	18.4
NUM	0.6	0.7	0.7	0.6
PRON	10.6	9.7	10.2	10.7
PROPN	4.3	4.9	4.2	4.2
PUNCT	19.8	19.7	19.6	20.1
SCONJ	2.5	2.3	2.5	2.6
SYM	0.4	0.0	0.6	0.4
VERB	15.9	16.1	16.3	15.9

### Lexical statistics

*Frequencies* For frequency statistics, we can clearly see that most words appear very infrequently (Table 6). Most of the word types observed in the entire TCBLex have SFI values between $SFI\approx 2$ and $SFI\approx 34$ (P10–P90), and the mean for $SFI\approx 15$, which means that the average word type is expected to occur less than once in 100 million tokens of written text. Similarly, the vast majority of word types have Zipf values below 3 (P10-P50 for ’7–8’, P10-P75 for ’9–12’ and ’13+’, and P10-P90 for TCBLex), which means they can be considered quite rare in their respective sub-lexicons.

**Fig. 2:**
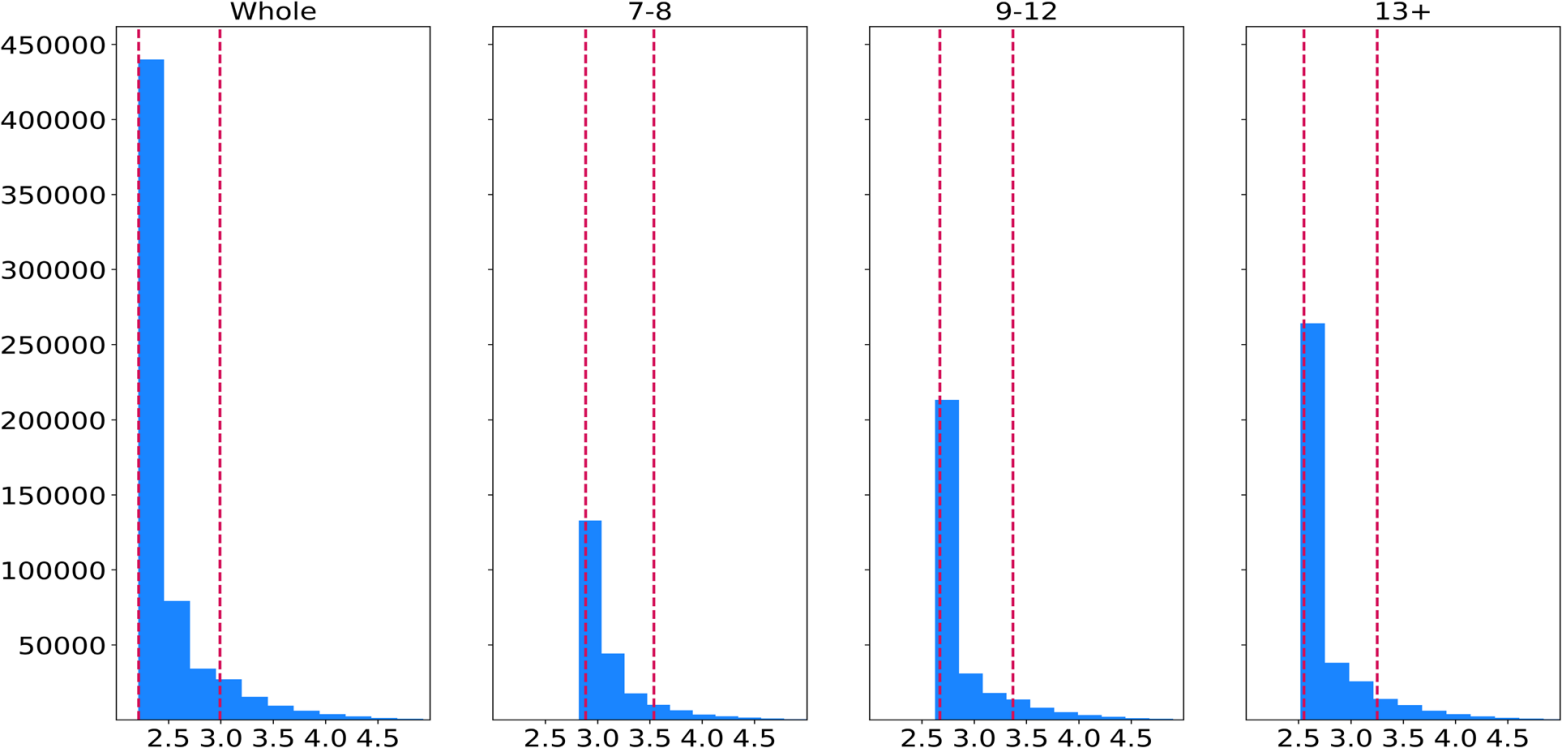
Zipf value frequencies of words for TCBLex 1.0, where the *x*-axes show Zipf values and the *y*-axes the frequency of the Zipf value in the specified sub-corpus. *Dotted lines* mark the 50th and 90th percentile for Zipf values

The maximum observed $SFI \approx 89$, which means that a word type or lemma is expected to occur around every ten tokens, belongs to the full stop punctuation mark. For non-punctuation symbols, the highest values are for words such as “ja” (Finnish for “and”, $SFI \approx 84$) and “on” (Finnish for “is”, $SFI \approx 80.4$). One can also look at the Zipf values of word types to see that the distribution of word types is quite similar across the age group-specific sub-lexicons, as well as the full lexical database (Fig. 2). This would seem to indicate that the frequencies in TCBLex are valid, as one can see the same Zipf distribution regardless of corpus size (van Heuven et al., 2014).

**Table 6:** Frequency and distribution statistics for TCBLex 1.0

	Word				Lemma			
	All	7–8	9–12	13+	All	7–8	9–12	13+
**F**								
M	18.68	10.781	13.328	14.262	47.642	27.006	35.126	37.3
SD	1715	587	839	1021	3078	1036	1528	1864
Min	1.0	1.0	1.0	1.0	1.0	1.0	1.0	1.0
P10	1.0	1.0	1.0	1.0	1.0	1.0	1.0	1.0
P25	1.0	1.0	1.0	1.0	1.0	1.0	1.0	1.0
P50	1.0	1.0	1.0	1.0	1.0	1.0	1.0	1.0
P75	3.0	3.0	3.0	3.0	4.0	4.0	4.0	4.0
P90	11.0	8.0	9.0	9.0	15.0	14.0	16.0	15.0
Max	989,683	204,742	339,772	445,169	989,683	204,742	339,772	445,169
**SFI**								
M	14.888	24.264	22.321	21.313	14.895	25.148	23.091	22.0
SD	13.321	10.884	11.297	11.245	13.995	12.035	12.389	12.221
Min	-9.46	4.27	0.415	3.708	-9.46	4.27	0.415	3.708
P10	2.106	14.015	12.085	10.408	1.929	13.807	12.085	10.408
P25	4.866	16.111	13.668	13.603	4.486	16.111	13.472	13.23
P50	8.365	19.684	16.773	15.725	8.341	19.797	16.942	16.14
P75	25.614	31.603	30.51	29.328	25.508	33.404	32.657	31.367
P90	34.478	40.51	38.925	38.027	35.729	43.017	41.422	40.009
Max	89.106	89.135	89.139	89.061	89.106	89.135	89.139	89.061
**Zipf**								
M	2.45	3.087	2.892	2.773	2.509	3.179	2.98	2.854
SD	0.415	0.368	0.388	0.391	0.496	0.468	0.489	0.487
Min	2.214	2.885	2.671	2.552	2.228	2.908	2.691	2.57
P10	2.214	2.885	2.671	2.552	2.228	2.908	2.691	2.57
P25	2.214	2.885	2.671	2.552	2.228	2.908	2.691	2.57
P50	2.214	2.885	2.671	2.552	2.228	2.908	2.691	2.57
P75	2.515	3.186	2.972	2.853	2.626	3.305	3.088	2.968
P90	2.992	3.538	3.37	3.251	3.131	3.783	3.62	3.473
Max	7.909	7.895	7.901	7.9	7.922	7.918	7.921	7.918
**CD**								
M	0.015	0.03	0.033	0.033	0.019	0.039	0.042	0.042
SD	0.053	0.072	0.076	0.078	0.072	0.101	0.106	0.105
Min	0.003	0.01	0.01	0.01	0.003	0.01	0.01	0.01
P10	0.003	0.01	0.01	0.01	0.003	0.01	0.01	0.01
P25	0.003	0.01	0.01	0.01	0.003	0.01	0.01	0.01
P50	0.003	0.01	0.01	0.01	0.003	0.01	0.01	0.01
P75	0.007	0.02	0.02	0.02	0.007	0.02	0.02	0.02
P90	0.023	0.05	0.06	0.06	0.027	0.07	0.08	0.08
Max	1.0	1.0	1.0	1.0	1.0	1.0	1.0	1.0

*Distribution* From the number of hapax words and lemmas, we could see that most words and lemmas appear only once. Looking at the CD values for word types and lemmas in TCBLex 1.0, we can see this clearly: Types showing in more than one book appear only in the 75th percentile. The mean CD for word types is around 0.015 and around 0.02 for lemmas, which translates to around five and seven books out of 300, respectively. This suggests that while the vast majority of words and lemmas appear only in one or two books (P10-P75), there are some words and lemmas that appear very often (Table 6.)

Looking at the data more closely, there are a total of 1950 word types that appear in at least half of the books, which means that only around 0.3% of all word types are encountered very frequently in different contexts. For lemmas, the number is slightly lower at 1648 types, but this represents around 0.7%, meaning that the proportion is higher for lemmas than for words. There are only 15 word types and 27 lemmas that occur in every single book. These include function words with very high SFI values, such as “ei” (Finnish for “no”) and the lemma “olla” (Finnish for “to be”), as well as slightly less frequent but very important lemmas like “päästä” (Finnish for “reach”). These word types and lemmas, despite being so few in number, account for a significant part of all text. The 15 word types with $CD = 1$ account for around $23\%$ of all words in the lexicon, and the 27 lemmas with $CD = 1$ account for over a third ($34\%$) of all tokens.

**Fig. 3:**
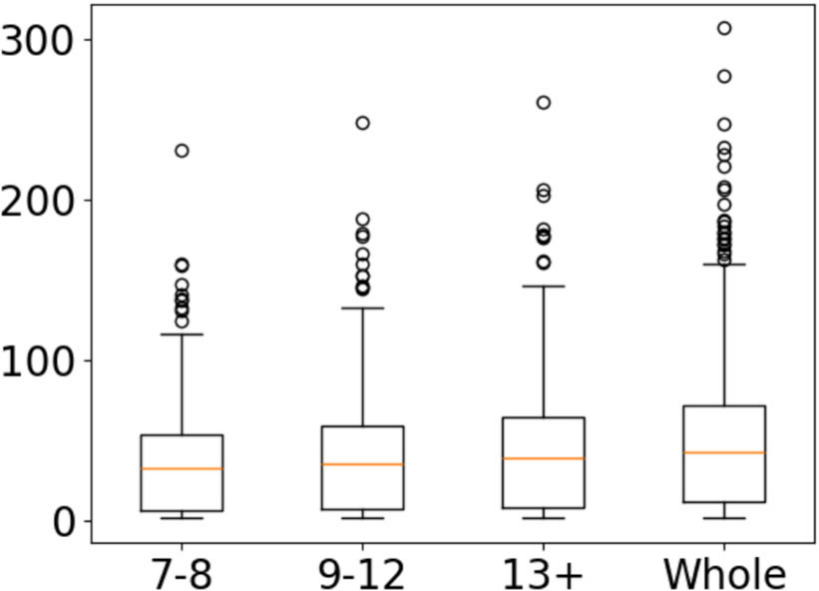
Morphological paradigm sizes for lemmas in the 90th percentile of absolute frequencies

*Morphological paradigm size* As can be expected, there are clear differences between age groups in terms of morphological paradigm size. Although most lemmas have only one or two forms, with the mean family size approximately 2.5 across the entire lexicon and the 50th percentile having only a single form, the effects can be seen most clearly with the most frequent lemmas (Fig. 3).

By far the largest family size belongs to the Finnish word “olla” (“to be” in English), and it has a total of 307 different forms inside the whole lexicon. Other lemmas with very large morphological paradigm sizes are “minä” (Finnish for “me”, $MPS=277$), “tehdä” (Finnish for “to do”, $MPS=247$), and “nähdä” (Finnish for “to see”, $MPS=233$). While a small number of these are undoubtedly lemmatizing mistakes, the results still follow those of Pajunen and Honko, where the family sizes of the most commonly used words grew in size as the ages of writers increased (Pajunen & Honko, 2021). The number of lemmas with $MPS=1$ is around 164, 000, which is larger than the number of hapax lemmas at around 138, 000. This shows that while there is significant overlap between lemmas which only occur once (hapax) and lemmas which always occur as the same word type ($MPS=1$), they are not equivalent.

*Word length and number of syllables* The average lengths of word types seem very similar across age groups in TCBLex 1.0. The mean lengths of word types for age groups are 10.1, 10.4, and 10.7 characters from youngest to oldest. The mean length for word types is around 11 characters in the entire lexicon (Table 7). On the token level, the mean length of words is significantly shorter, around 5.3 characters, although this is skewed by high-frequency, one-character tokens, such as full stops and commas. In fact, word types with a length lower than six characters make up around 55% of all tokens in the lexicon. If we ignore tokens with length one, which alone make up about 20% of all tokens, then the average word length for TCBLex 1.0 is slightly longer, around 6.4 characters. On the token level, there seem to be no differences in mean word lengths between age groups.

**Table 7:** Length statistics of TCBLex 1.0 for word types and tokens, excluding types with length of one character

	Word Types				Tokens			
	All	7-8	9-12	13+	All	7-8	9-12	13+
M	11	10.1	10.4	10.7	6.5	6.4	6.4	6.4
SD	3.7	3.4	3.5	3.6	3.1	3.1	3.1	3.1
Min	2	2	2	2	2	2	2	2
P10	7	6	6	6	3	3	3	3
P25	8	8	8	8	4	4	4	4
P50	11	10	10	10	6	6	6	6
P75	13	12	12	13	8	8	8	8
P90	16	14	15	15	11	10	11	11
Max	69	69	42	50	69	69	42	50

**Table 8:** Statistics on the number of syllables per word type in TCBLex 1.0

	All	7–8	9–12	13+
M	4.4	4.1	4.2	4.3
SD	1.5	1.4	1.4	1.5
Min	0	0	0	0
P10	3	2	3	3
P25	3	3	3	3
P50	4	4	4	4
P75	5	5	5	5
P90	6	6	6	6
Max	26	26	17	17

There seems to be a small increase in the average length of word types as the intended reading age increases, but the difference is modest. These results are similar to previous results showing that words tend to increase in length as the target age increases, albeit slightly (Lété et al., 2004; Schroeder et al., 2015; Terzopoulos et al., 2016). This slight increase in word length when the intended reading age increases can also be seen with the number of syllables per word (Table 8). However, the mean number of syllables is very similar for all sub-lexicons, with around four syllables per word type.

**Fig. 4:**
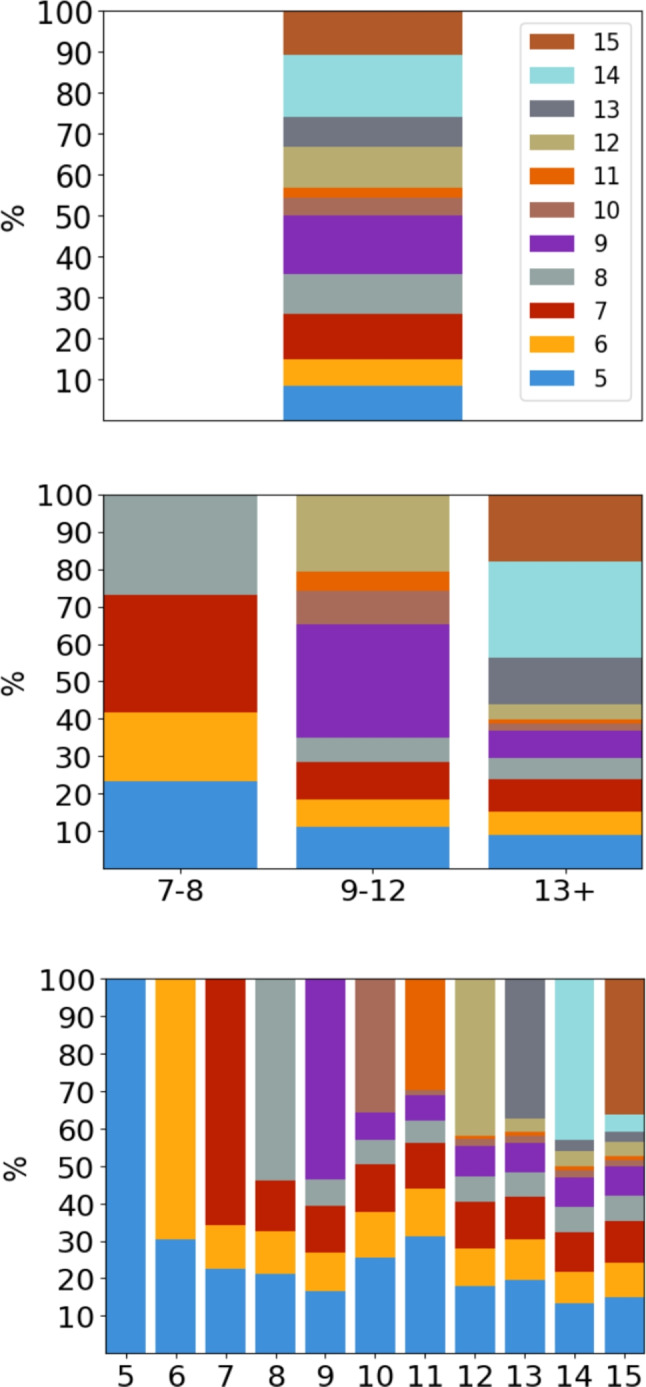
Proportions of how many word types are first seen at what age for TCBLex 1.0

The maximum observed length is for a made-up chemical term in the age group ‘7–8’ with a length of 69 characters. The second longest word, and the one with the highest number of syllables, is also found in the youngest age group: “seitsemällätuhannellayhdeksälläsadallakahdeksallakymmenelläkolmella” (Finnish for the number 7983 written out in the adessive case), which has a length of 67 characters and has 26 syllables.

*FSA values* One of the most interesting findings from TCBLex 1.0 is that many word types are age or age group specific and only a relatively small portion of word types carry over as the intended reading age of books increases (Fig. 4). As can be seen when looking at the distribution of the ages in which word types are first encountered, it matches very closely the distribution of books sampled for TCBLex 1.0 (Fig. 1). This would suggest that a large number of word types are, most likely, book-specific, which is further validated if looking at the distribution of first seen at ages (FSA) over age-specific sub-lexicons. Intuitively, this makes sense, as if books are not a part of the same series, proper nouns like character names usually do not carry over from book to book. Despite this, there is still a clear trend of word types getting added to the general vocabulary inside children’s books as the intended reading age increases. For example, about $44\%$ of the word types in ’13+’ are encountered in books meant for younger children. On the token level, around 79% of all tokens in the lexicon are encountered in books meant for 5-year-olds, which means that the most commonly used words in written Finnish are encountered very early on. So, for example, where around 10% of the word types in both age groups ’9–12’ and ’13+’ are first encountered in books intended to be read by 5-year-olds, these word types make up around 80% of the total amount of tokens in the books for these age groups. And for all intended reading ages past 8, the word types first encountered for a particular age make less than $10\%$ of the tokens for that sub-lexicon.

## Conclusion

This work introduces TCBLex 1.0, the first Finnish lexical database of children’s books. It consists of 300 books and has information on the level of intended reading ages, containing 14 different sub-lexicons, covering individual ages, age groups, and different genres. TCBLex 1.0 joins a growing list of lexical databases of children’s literary works (see Table 1) and exhibits similar findings for Finnish as have been found for other languages. The most striking is the number of hapax words and lemmas, with them making up around 58% of all word types and around 56% lemmas found in TCBLex 1.0. Although other works have also reported high amounts of hapax words, thus far the highest proportions were found in German with around 48% (Schroeder et al., 2015). As the number of hapax types is very high with both words and lemmas, this would suggest that children reading Finnish books encounter very many new words on a constant basis and that there is perhaps even less lexical overlap between books than in other languages. The SFI and Zipf values also point to the vast majority of words and lemmas being very rare, with the mean expected frequency for word types being once in 100 million. However, one has to keep in mind that while the number of hapax types is very high and a lot of words are very rare, there are also a lot of very common words, with 15 word types with the highest CD making up around $23\%$ of all tokens in TCBLex 1.0.

We believe TCBLex will make an impact in the Finnish psycholinguistics field, as it is the first lexical database of Finnish literary works that also reports information on what intended reading age word types and lemmas are first seen at. It is also substantial in size with around 11.6 million tokens, which is quite large in the Finnish context. This means that, for example, TCBLex can be used in the selection of appropriate stimuli words for Finnish psycholinguistics research of reading development. TCBLex 1.0 is freely available as a collection of CSV files. The GitHub page has documentation on how to interpret the CSV files, and we also provide the Python notebooks used to query TCBLex, which have been used to provide the statistics reported in this paper. Modifying the notebooks is quite straightforward, and it should be easy to add functionality to, for example, calculate OLD20 values (Yarkoni et al., 2008) for word types.

### Limitations and future work

One of the greatest limitations of our study is the accuracy of the tools we used. Trankit performs very well in many tasks, but the lemmatization accuracy in specific is not perfect for Finnish (see the Processing pipeline section). To make sure the lemma data in our corpus was within the expected error rates, we checked if the numbers reported on Trankit’s documentation page were reliable by performing manual annotations on a test sample and checking our results against Trankit’s output. We randomly selected a sample of 300 sentences from TCBC, containing a total of 2817 tokens, and compared our annotations to those made by Trankit. This gave Trankit a lemmatization accuracy of around 96% for all tokens and a 95% accuracy if we excluded non-words, such as periods. Most of the mistakes appeared to be incorrectly identifying base words used to construct compounds and with inflections of proper nouns, particularly if the word was foreign. This would suggest that the accuracies reported by Trankit are indeed reliable, which also means that, e.g., the statistics for hapax lemmas are likely slightly different in reality. In the future, we will look to test other tools or different methods to gain better lemmatization and tagging accuracies, providing even higher-quality data in future versions of the lexicon.

A potential bias worth mentioning is how the FSA values are assigned. As we have only chosen a sample of children’s books, the values are inherently tied to which books are included in our corpus. In addition, while a vast majority of the books on our list had an exact intended reading age, our choice to choose the lowest possible age when a reading age was given as a span produces slightly lower FSA values than if we had made a different choice.

In the future, we will also look to expand the corpus TCBLex is based on. We already have plans to increase the number of textbooks for each age group by at least a factor of two, so that we can have a sizable subcorpus of just textbooks and better represent the fact that a lot of children’s reading comes in the form of school materials. As part of this, we are aiming to grow the total size of the corpus from 300 books to at least 525 books and to increase the number of books from those intended reading ages that have very few books in the current version, like 11. We also plan to use the corpus TCBLex is based on in future studies revolving around syntactic complexity beyond the lexical level and what sorts of syntactic structures can be used to identify suitable textual materials for certain age groups.

## Data Availability

The lexical database is available at: 10.5281/zenodo.15655580.

## Declarations

### Code availability

The code used is available at: https://github.com/TurkuNLP/TCBLex.
