# Author Correction: TCBLex - A lexical database of Finnish literary texts for children

**DOI:** 10.3758/s13428-026-03062-5

**Published:** 2026-07-07

**Authors:** Tapio Nojonen, Kiia Korsu, Filip Ginter, Veronika Laippala, Jenna Kanerva

**Affiliations:** 1 Department of Computing, University of Turku, Turku, Southwest Finland, FI Finland; 2 Department of Biology, University of Turku, Turku, Southwest Finland, FI Finland; 3 School of Languages and Translations Studies, University of Turku, Turku, Southwest Finland, FI Finland

**Author Correction: Behavior Research Methods (2025) 57:312**

10.3758/s13428-025-02832-x

The original online version of this article was revised: The numbers in Table 8 were incorrect and have been updated.

'All', 5.2 became 4.4; 1.4 became 1.5; 9.0 became 0; 7.0 became 3; 4.0 became 3; 0.4 became 0; 18.8 became 5; 0.6 became 6; 10.6 became 26.

In the column '7-8', 5.6 became 4.1; 9.1 became 0; 6.4 became 2; 3.9 became 3; 0.4 became 4; 19.5 became 5; 0.7 became 6; 9.7 became 26.

In the column '9-12', 5.1 became 4.2; 9.2 became 0; 6.8 became 3; 3.8 became 3; 0.3 became 4; 19.1 became 5; 0.7 became 6; 10.2 became 17.

In the column '13+', 5.2 became 4.3; 1.4 became 1.5; 8.9 became 0; 7.1 became 3; 4.0 became 3; 0.4 became 4; 18.4 became 5; 0.6 became 6; 10.7 became 17.

